# 7-Benzyl-3-(4-fluoro­phen­yl)-2-propyl­amino-5,6,7,8-tetra­hydro­pyrido[4′,3′:4,5]thieno[2,3-*d*]pyrimidin-4(3*H*)-one

**DOI:** 10.1107/S1600536812010318

**Published:** 2012-03-17

**Authors:** Hai-Jun Hu, Hong Chen

**Affiliations:** a Hubei Key Laboratory of Natural Products Research and Development, China Three Gorges University, Yichang 443002, People’s Republic of China; b College of Chemistry and Life Science, China Three Gorges University, Yichang 443002, People’s Republic of China

## Abstract

In the title compound, C_25_H_25_FN_4_OS, the thienopyrimidine fused-ring system is close to planar (r.m.s. deviation = 0.0089 Å), with a maximum deviation of 0.0261 (17) Å for the N atom adjacent to the benzene ring. This thienopyrimidine fused-ring system forms dihedral angles of 64.73 (3) and 81.56 (5)° with the adjacent benzyl and fluoro­phenyl rings, respectively. Inter­molecular N—H⋯F and C—H⋯F hydrogen bonding, as well as C—F⋯π inter­actions [F⋯centroid = 3.449 (3) Å; C—F⋯centroid = 91.87 (15)°], help to stabilize the crystal structure.

## Related literature

For the biological and pharmaceutical properties of compounds containing the fused thienopyrimidine system, see: Amr *et al.* (2010 ▶); Huang *et al.* (2009 ▶); Mavrova *et al.* (2010 ▶). For similar crystal structures, see: Xie *et al.* (2008) ▶; Chen *et al.* (2011 ▶). 
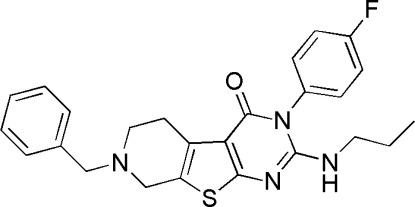


## Experimental

### 

#### Crystal data

C_25_H_25_FN_4_OS
*M* *_r_* = 448.55
Orthorhombic, 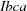

*a* = 17.921 (7) Å
*b* = 18.427 (7) Å
*c* = 27.114 (10) Å
*V* = 8954 (6) Å^3^
*Z* = 16
Mo *K*α radiation
μ = 0.18 mm^−1^
*T* = 296 K
0.26 × 0.25 × 0.23 mm

#### Data collection

Bruker SMART CCD diffractometer
Absorption correction: multi-scan (*SADABS*; Sheldrick, 1996 ▶) *T*_min_ = 0.955, *T*_max_ = 0.960
46089 measured reflections
5152 independent reflections
4234 reflections with *I* > 2σ(*I*)
*R*_int_ = 0.107

#### Refinement

*R*[*F* ^2^ > 2σ(*F* ^2^)] = 0.069
*wR*(*F* ^2^) = 0.194
*S* = 1.05
5152 reflections
290 parameters
H-atom parameters constrained
Δρ_max_ = 0.58 e Å^−3^
Δρ_min_ = −0.30 e Å^−3^

### 

Data collection: *SMART* (Bruker, 1997 ▶); cell refinement: *SAINT* (Bruker, 1997 ▶); data reduction: *SAINT*; program(s) used to solve structure: *SHELXTL* (Sheldrick, 2008 ▶); program(s) used to refine structure: *SHELXTL*; molecular graphics: *SHELXTL*; software used to prepare material for publication: *SHELXTL*.

## Supplementary Material

Crystal structure: contains datablock(s) I, global. DOI: 10.1107/S1600536812010318/gw2115sup1.cif

Structure factors: contains datablock(s) I. DOI: 10.1107/S1600536812010318/gw2115Isup2.hkl

Supplementary material file. DOI: 10.1107/S1600536812010318/gw2115Isup3.cml

Additional supplementary materials:  crystallographic information; 3D view; checkCIF report

## Figures and Tables

**Table 1:** Hydrogen-bond geometry (Å, °)

*D*—H⋯*A*	*D*—H	H⋯*A*	*D*⋯*A*	*D*—H⋯*A*
C24—H24*B*⋯F1^i^	0.97	2.66	3.258 (5)	121
C25—H25*A*⋯F1^i^	0.96	2.56	3.096 (5)	116
N4—H4*A*⋯F1^i^	0.86	2.65	3.423 (3)	151

Symmetry code: (i) 
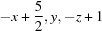
.

**Fig. 1.**
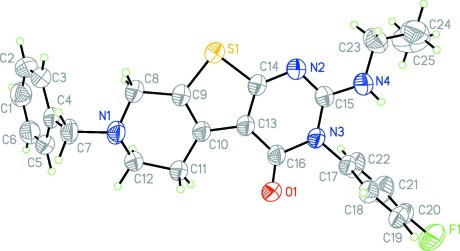
Molecular structure of the title compound with 50% probability displacement ellipsoids.

**Fig. 2.**
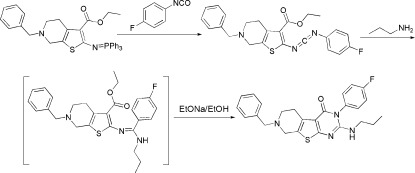
Reaction scheme.

## supplementary crystallographic information

### Comment

Derivatives of heterocycles containing the thienopyrimidine system have proved to show significant antifungal, antibacterical, anticonvulsant and angiotensin antagonistic activities (Amr *et al.* 2010; Huang *et al.* 2009; Mavrova *et al.* 2010). Recently, we have focused on the synthesis of fused heterocyclic systems containing thienopyrimidine *via* aza-wittig reaction under mild conditions. Some X-ray crystal structures of fused pyrimidinone derivatives have been reported (Chen *et al.*, 2011; Xie *et al.*, 2008). The title compound has potential use as a precursor for obtaining bioactive molecules with fluorescence properties. Herein, we report its crystal structure (Fig. 1).

In the crystal structure of the title compound, C_25_H_25_FN_4_OS, the thienopyrimidine fused-ring system is close to coplanar (r.m.s deviation = 0.0089 Å) with a maximum deviation of -0.0261 (17) Å for atom N(3). This ring system forms diherdral angles of 64.73 (3) and 81.56 (5)° with the adjacent 6-membered rings C1—C6 and C17—C22, respectively. Most bond lengths in the system fell in the range of single and double bonds, for example, the bond lengths of C(9)—C(10), C(13)—C(14) and C(16)—O(1) were in accordance with the double bond distances. Intermolecular N—H···F (N4—H4A···F1^i^ with symmetry code: (i) 3/2 - *x*, *y*, -*z*) and C—H···F hydrogen bonding (C24—H24B···F1^i^ and C25—H25A···F1^i^), as well as C—F···π interactions (C20—F1···*Cg*1 with *Cg*1 centroids of the C17—C18—C19—C20—C21 ring), helps to stabilize the crystal structure.

### Experimental

1-fluoro-4-isocyanatobenzene (2 mmol) under nitrogen atmosphere was added to a solution of iminophosphorane (2 mmol) in anhydrous CH_2_Cl_2_ (10 ml) at room temperature. When the reaction mixture was left unstirred for 12 h at 273–278 k, iminophosphorane was consumed (TLC monitored). The solvent was removed under reduced pressure and ether/petroleum ether (volume ratio 1:2, 20 ml) was added to precipitate triphenylphosphine oxide. Removal of the solvent gave carbodiimide, which was used directly without further purification. Propan-1-amine (2 mmol) was added to the solution of carbodiimide in anhydrous dichloromethane (10 ml). After the reaction mixture was left unstirred for 5–6 h, the solvent was removed and anhydrous EtOH (10 ml) with several drops of EtONa (in EtOH) was added to the mixture. The mixture was stirred for another 6–8 h at room temperature. The solution was condensed and the residual was recrystallized from EtOH to give the expected title compound as white crystals, 0.832 g (87%), *M*.p. 431–432 K; ^1^H NMR (CDCl_3_, 600 MHz) *δ*: 7.37–7.25 (m, 9H, Ar—H), 4.06 (br, 1H, NH), 3.72 (s, 2H, Ar—CH_2_), 3.59 (s, 2H, NCH_2_-thiophene), 3.31 (m, 2H, NHCH_2_), 2.98 (t, *J* = 5.1 Hz, 2H, NCH_2_CH_2_), 2.82 (t, *J* = 5.1 Hz, 2H, NCH_2_CH_2_), 1.50–1.48 (m, 2H, CH_2_CH_3_), 0.84 (t, *J* = 6.6 Hz, 3H, CH_2_CH_3_); IR (KBr) *v*: 3373 (N—H), 1673 (C=O), 1540, 1378, 696 cm^-1^; EI—MS *m/z* (%): 448 (*M*^+^, 15), 357 (16), 329 (100), 287 (26), 91 (72). Anal. calcd for C_25_H_25_FN_4_OS: C 66.94, H 5.62, N 12.49; found: C 66.71, H 5.50, N 12.23.

### Refinement

All H atoms were positioned geometrically [C—H = 0.93, 0.96, 0.97 Å and N—H = 0.86 Å] and allowed to ride on their parent atoms, with *U*_iso_(H) = 1.2*U*_eq_(C, N).

### Crystal data

**Table:**

C_25_H_25_FN_4_OS	*F*(000) = 3776
*M**_r_* = 448.55	*D*_x_ = 1.331 Mg m^−^^3^
Orthorhombic, *I**b**c**a*	Melting point: 432 K
Hall symbol: -I 2b 2c	Mo *K*α radiation, λ = 0.71073 Å
*a* = 17.921 (7) Å	θ = 2.2–27.5°
*b* = 18.427 (7) Å	µ = 0.18 mm^−^^1^
*c* = 27.114 (10) Å	*T* = 296 K
*V* = 8954 (6) Å^3^	Block, white
*Z* = 16	0.26 × 0.25 × 0.23 mm

### Data collection

**Table:**

Bruker SMART CCD diffractometer	5152 independent reflections
Radiation source: fine-focus sealed tube	4234 reflections with *I* > 2σ(*I*)
Graphite monochromator	*R*_int_ = 0.107
CCD Profile fitting scans	θ_max_ = 27.5°, θ_min_ = 2.2°
Absorption correction: multi-scan (*SADABS*; Sheldrick, 1996)	*h* = −23→23
*T*_min_ = 0.955, *T*_max_ = 0.960	*k* = −23→23
46089 measured reflections	*l* = −35→35

### Refinement

**Table:**

Refinement on *F*^2^	Primary atom site location: structure-invariant direct methods
Least-squares matrix: full	Secondary atom site location: difference Fourier map
*R*[*F*^2^ > 2σ(*F*^2^)] = 0.069	Hydrogen site location: inferred from neighbouring sites
*wR*(*F*^2^) = 0.194	H-atom parameters constrained
*S* = 1.05	*w* = 1/[σ^2^(*F*_o_^2^) + (0.090*P*)^2^ + 9.9439*P*] where *P* = (*F*_o_^2^ + 2*F*_c_^2^)/3
5152 reflections	(Δ/σ)_max_ = 0.001
290 parameters	Δρ_max_ = 0.58 e Å^−^^3^
0 restraints	Δρ_min_ = −0.30 e Å^−^^3^

### Special details

**Table:**

Geometry. All e.s.d.'s (except the e.s.d. in the dihedral angle between two l.s. planes) are estimated using the full covariance matrix. The cell e.s.d.'s are taken into account individually in the estimation of e.s.d.'s in distances, angles and torsion angles; correlations between e.s.d.'s in cell parameters are only used when they are defined by crystal symmetry. An approximate (isotropic) treatment of cell e.s.d.'s is used for estimating e.s.d.'s involving l.s. planes.
Refinement. Refinement of *F*^2^ against ALL reflections. The weighted *R*-factor *wR* and goodness of fit *S* are based on *F*^2^, conventional *R*-factors *R* are based on *F*, with *F* set to zero for negative *F*^2^. The threshold expression of *F*^2^ > σ(*F*^2^) is used only for calculating *R*-factors(gt) *etc*. and is not relevant to the choice of reflections for refinement. *R*-factors based on *F*^2^ are statistically about twice as large as those based on *F*, and *R*- factors based on ALL data will be even larger.

### Fractional atomic coordinates and isotropic or equivalent isotropic displacement parameters (Å^2^)

**Table:**

	*x*	*y*	*z*	*U*_iso_*/*U*_eq_	
S1	0.87109 (4)	0.17415 (4)	0.30237 (2)	0.0591 (2)	
C1	0.46974 (17)	0.04644 (18)	0.32327 (13)	0.0698 (8)	
H1	0.4418	0.0066	0.3132	0.084*	
C2	0.50154 (17)	0.09137 (18)	0.28892 (11)	0.0674 (8)	
H2	0.4948	0.0820	0.2555	0.081*	
C3	0.54325 (15)	0.15022 (16)	0.30340 (10)	0.0571 (6)	
H3	0.5640	0.1805	0.2796	0.068*	
C4	0.55497 (13)	0.16516 (14)	0.35321 (9)	0.0496 (6)	
C5	0.52218 (15)	0.11933 (16)	0.38753 (10)	0.0587 (7)	
H5	0.5290	0.1282	0.4210	0.070*	
C6	0.47947 (17)	0.06071 (18)	0.37275 (12)	0.0673 (8)	
H6	0.4573	0.0309	0.3962	0.081*	
C7	0.60106 (14)	0.22989 (15)	0.36911 (11)	0.0583 (7)	
H7A	0.5969	0.2669	0.3438	0.070*	
H7B	0.5792	0.2495	0.3990	0.070*	
C8	0.72121 (13)	0.19589 (15)	0.33405 (10)	0.0530 (6)	
H8A	0.7181	0.2345	0.3098	0.064*	
H8B	0.6990	0.1526	0.3200	0.064*	
C9	0.80136 (13)	0.18162 (14)	0.34679 (9)	0.0495 (6)	
C10	0.82718 (13)	0.17246 (13)	0.39342 (9)	0.0452 (5)	
C11	0.77549 (13)	0.17373 (15)	0.43661 (9)	0.0522 (6)	
H11A	0.7811	0.2191	0.4543	0.063*	
H11B	0.7877	0.1343	0.4589	0.063*	
C12	0.69484 (14)	0.16574 (15)	0.41885 (9)	0.0520 (6)	
H12A	0.6863	0.1165	0.4076	0.062*	
H12B	0.6608	0.1756	0.4458	0.062*	
C13	0.90652 (12)	0.16038 (12)	0.39418 (9)	0.0452 (5)	
C14	0.93774 (13)	0.15967 (14)	0.34782 (9)	0.0492 (6)	
C15	1.05433 (13)	0.13502 (14)	0.37363 (9)	0.0513 (6)	
C16	0.95394 (12)	0.14756 (13)	0.43557 (9)	0.0445 (5)	
C17	1.07929 (12)	0.11374 (13)	0.46200 (9)	0.0444 (5)	
C18	1.11037 (14)	0.16664 (13)	0.49144 (10)	0.0507 (6)	
H18	1.0994	0.2153	0.4859	0.061*	
C19	1.15808 (14)	0.14701 (16)	0.52937 (10)	0.0574 (6)	
H19	1.1794	0.1819	0.5498	0.069*	
C20	1.17280 (15)	0.07522 (17)	0.53586 (10)	0.0605 (7)	
C21	1.14149 (17)	0.02195 (16)	0.50825 (13)	0.0688 (8)	
H21	1.1518	−0.0266	0.5147	0.083*	
C22	1.09393 (16)	0.04143 (14)	0.47039 (12)	0.0615 (7)	
H22	1.0720	0.0060	0.4507	0.074*	
C23	1.15905 (18)	0.1213 (2)	0.31582 (13)	0.0810 (10)	
H23A	1.1625	0.1710	0.3043	0.097*	
H23B	1.1256	0.0955	0.2938	0.097*	
C24	1.2338 (2)	0.0875 (2)	0.31314 (14)	0.0900 (11)	
H24A	1.2548	0.0963	0.2807	0.108*	
H24B	1.2662	0.1104	0.3372	0.108*	
C25	1.2322 (3)	0.0086 (2)	0.32243 (17)	0.1213 (17)	
H25A	1.2107	−0.0005	0.3542	0.182*	
H25B	1.2822	−0.0102	0.3216	0.182*	
H25C	1.2028	−0.0149	0.2975	0.182*	
N1	0.68111 (11)	0.21671 (11)	0.37846 (8)	0.0519 (5)	
N2	1.01070 (11)	0.14831 (13)	0.33586 (8)	0.0560 (5)	
N3	1.02934 (10)	0.13358 (11)	0.42237 (7)	0.0471 (5)	
N4	1.12699 (12)	0.12111 (16)	0.36574 (9)	0.0667 (7)	
H4A	1.1554	0.1119	0.3905	0.080*	
O1	0.93579 (10)	0.14699 (11)	0.47917 (6)	0.0568 (5)	
F1	1.22046 (12)	0.05586 (13)	0.57255 (7)	0.0979 (7)	

### Atomic displacement parameters (Å^2^)

**Table:**

	*U*^11^	*U*^22^	*U*^33^	*U*^12^	*U*^13^	*U*^23^
S1	0.0455 (4)	0.0870 (5)	0.0447 (4)	0.0112 (3)	−0.0047 (3)	0.0065 (3)
C1	0.0575 (16)	0.0736 (19)	0.078 (2)	−0.0005 (14)	−0.0060 (15)	−0.0112 (16)
C2	0.0616 (16)	0.090 (2)	0.0500 (15)	0.0039 (16)	−0.0082 (13)	−0.0146 (14)
C3	0.0490 (13)	0.0763 (17)	0.0460 (13)	0.0092 (12)	−0.0013 (11)	0.0003 (12)
C4	0.0362 (11)	0.0627 (14)	0.0499 (13)	0.0181 (10)	−0.0067 (10)	−0.0054 (11)
C5	0.0524 (14)	0.0802 (18)	0.0435 (13)	0.0193 (13)	−0.0041 (11)	−0.0007 (12)
C6	0.0564 (16)	0.0786 (19)	0.0668 (18)	0.0092 (14)	0.0029 (14)	0.0103 (15)
C7	0.0418 (12)	0.0654 (16)	0.0677 (17)	0.0201 (12)	−0.0108 (12)	−0.0123 (13)
C8	0.0432 (12)	0.0642 (15)	0.0516 (14)	0.0114 (11)	−0.0092 (11)	0.0023 (11)
C9	0.0403 (12)	0.0592 (14)	0.0489 (13)	0.0082 (10)	−0.0050 (10)	0.0013 (11)
C10	0.0392 (11)	0.0506 (12)	0.0457 (12)	0.0101 (9)	−0.0050 (9)	−0.0048 (10)
C11	0.0420 (12)	0.0700 (16)	0.0447 (13)	0.0140 (11)	−0.0056 (10)	−0.0060 (11)
C12	0.0425 (12)	0.0669 (15)	0.0465 (13)	0.0120 (11)	−0.0024 (10)	−0.0080 (11)
C13	0.0380 (11)	0.0518 (12)	0.0457 (12)	0.0073 (9)	−0.0039 (9)	−0.0040 (10)
C14	0.0401 (11)	0.0596 (14)	0.0478 (13)	0.0083 (10)	−0.0062 (10)	0.0014 (11)
C15	0.0398 (12)	0.0647 (15)	0.0493 (13)	0.0093 (11)	0.0002 (10)	−0.0035 (11)
C16	0.0363 (11)	0.0488 (12)	0.0484 (13)	0.0071 (9)	−0.0037 (9)	−0.0081 (10)
C17	0.0348 (10)	0.0525 (13)	0.0458 (12)	0.0037 (9)	−0.0066 (9)	−0.0040 (10)
C18	0.0453 (12)	0.0495 (13)	0.0572 (15)	0.0005 (10)	−0.0047 (11)	−0.0053 (11)
C19	0.0449 (13)	0.0731 (17)	0.0542 (15)	−0.0050 (12)	−0.0077 (11)	−0.0152 (13)
C20	0.0452 (13)	0.088 (2)	0.0485 (14)	0.0082 (13)	−0.0105 (11)	0.0052 (13)
C21	0.0661 (17)	0.0546 (15)	0.086 (2)	0.0117 (13)	−0.0186 (16)	0.0080 (15)
C22	0.0564 (15)	0.0500 (14)	0.0780 (19)	0.0051 (12)	−0.0196 (14)	−0.0104 (13)
C23	0.0612 (18)	0.112 (3)	0.070 (2)	0.0229 (18)	0.0134 (16)	0.0164 (19)
C24	0.083 (2)	0.121 (3)	0.066 (2)	0.025 (2)	0.0101 (18)	0.0016 (19)
C25	0.179 (5)	0.092 (3)	0.094 (3)	0.036 (3)	−0.002 (3)	−0.014 (2)
N1	0.0415 (10)	0.0570 (12)	0.0572 (12)	0.0142 (9)	−0.0088 (9)	−0.0070 (9)
N2	0.0419 (10)	0.0796 (15)	0.0465 (11)	0.0110 (10)	−0.0011 (9)	0.0005 (10)
N3	0.0370 (9)	0.0595 (12)	0.0448 (10)	0.0102 (8)	−0.0067 (8)	−0.0066 (9)
N4	0.0412 (11)	0.1052 (19)	0.0538 (13)	0.0170 (12)	−0.0007 (9)	0.0013 (13)
O1	0.0460 (9)	0.0815 (13)	0.0428 (9)	0.0111 (9)	−0.0057 (7)	−0.0078 (8)
F1	0.0855 (13)	0.1396 (19)	0.0686 (12)	0.0214 (13)	−0.0338 (10)	0.0128 (11)

### Geometric parameters (Å, º)

**Table:**

S1—C14	1.737 (2)	C13—C16	1.427 (3)
S1—C9	1.741 (3)	C14—N2	1.363 (3)
C1—C2	1.370 (5)	C15—N2	1.312 (3)
C1—C6	1.378 (5)	C15—N4	1.344 (3)
C1—H1	0.9300	C15—N3	1.395 (3)
C2—C3	1.374 (4)	C16—O1	1.226 (3)
C2—H2	0.9300	C16—N3	1.421 (3)
C3—C4	1.394 (4)	C17—C18	1.377 (3)
C3—H3	0.9300	C17—C22	1.377 (3)
C4—C5	1.387 (4)	C17—N3	1.446 (3)
C4—C7	1.513 (4)	C18—C19	1.385 (4)
C5—C6	1.383 (4)	C18—H18	0.9300
C5—H5	0.9300	C19—C20	1.360 (4)
C6—H6	0.9300	C19—H19	0.9300
C7—N1	1.477 (3)	C20—F1	1.359 (3)
C7—H7A	0.9700	C20—C21	1.356 (4)
C7—H7B	0.9700	C21—C22	1.382 (4)
C8—N1	1.454 (3)	C21—H21	0.9300
C8—C9	1.501 (3)	C22—H22	0.9300
C8—H8A	0.9700	C23—N4	1.471 (4)
C8—H8B	0.9700	C23—C24	1.479 (5)
C9—C10	1.357 (3)	C23—H23A	0.9700
C10—C13	1.439 (3)	C23—H23B	0.9700
C10—C11	1.493 (3)	C24—C25	1.476 (6)
C11—C12	1.531 (3)	C24—H24A	0.9700
C11—H11A	0.9700	C24—H24B	0.9700
C11—H11B	0.9700	C25—H25A	0.9600
C12—N1	1.464 (3)	C25—H25B	0.9600
C12—H12A	0.9700	C25—H25C	0.9600
C12—H12B	0.9700	N4—H4A	0.8600
C13—C14	1.376 (3)		
			
C14—S1—C9	90.85 (12)	C13—C14—S1	111.54 (18)
C2—C1—C6	119.6 (3)	N2—C15—N4	119.3 (2)
C2—C1—H1	120.2	N2—C15—N3	123.5 (2)
C6—C1—H1	120.2	N4—C15—N3	117.3 (2)
C3—C2—C1	120.6 (3)	O1—C16—N3	119.6 (2)
C3—C2—H2	119.7	O1—C16—C13	127.0 (2)
C1—C2—H2	119.7	N3—C16—C13	113.5 (2)
C2—C3—C4	121.0 (3)	C18—C17—C22	120.8 (2)
C2—C3—H3	119.5	C18—C17—N3	120.1 (2)
C4—C3—H3	119.5	C22—C17—N3	119.0 (2)
C5—C4—C3	117.8 (3)	C17—C18—C19	119.7 (2)
C5—C4—C7	121.3 (2)	C17—C18—H18	120.2
C3—C4—C7	120.9 (3)	C19—C18—H18	120.2
C6—C5—C4	121.0 (3)	C20—C19—C18	118.0 (2)
C6—C5—H5	119.5	C20—C19—H19	121.0
C4—C5—H5	119.5	C18—C19—H19	121.0
C1—C6—C5	120.1 (3)	F1—C20—C21	118.3 (3)
C1—C6—H6	120.0	F1—C20—C19	118.2 (3)
C5—C6—H6	120.0	C21—C20—C19	123.5 (2)
N1—C7—C4	116.7 (2)	C20—C21—C22	118.5 (3)
N1—C7—H7A	108.1	C20—C21—H21	120.7
C4—C7—H7A	108.1	C22—C21—H21	120.7
N1—C7—H7B	108.1	C21—C22—C17	119.5 (2)
C4—C7—H7B	108.1	C21—C22—H22	120.3
H7A—C7—H7B	107.3	C17—C22—H22	120.3
N1—C8—C9	109.2 (2)	N4—C23—C24	113.5 (3)
N1—C8—H8A	109.8	N4—C23—H23A	108.9
C9—C8—H8A	109.8	C24—C23—H23A	108.9
N1—C8—H8B	109.8	N4—C23—H23B	108.9
C9—C8—H8B	109.8	C24—C23—H23B	108.9
H8A—C8—H8B	108.3	H23A—C23—H23B	107.7
C10—C9—C8	124.2 (2)	C25—C24—C23	112.9 (4)
C10—C9—S1	112.95 (18)	C25—C24—H24A	109.0
C8—C9—S1	122.80 (18)	C23—C24—H24A	109.0
C9—C10—C13	111.7 (2)	C25—C24—H24B	109.0
C9—C10—C11	121.1 (2)	C23—C24—H24B	109.0
C13—C10—C11	127.2 (2)	H24A—C24—H24B	107.8
C10—C11—C12	109.7 (2)	C24—C25—H25A	109.5
C10—C11—H11A	109.7	C24—C25—H25B	109.5
C12—C11—H11A	109.7	H25A—C25—H25B	109.5
C10—C11—H11B	109.7	C24—C25—H25C	109.5
C12—C11—H11B	109.7	H25A—C25—H25C	109.5
H11A—C11—H11B	108.2	H25B—C25—H25C	109.5
N1—C12—C11	109.4 (2)	C8—N1—C12	111.55 (19)
N1—C12—H12A	109.8	C8—N1—C7	112.4 (2)
C11—C12—H12A	109.8	C12—N1—C7	113.4 (2)
N1—C12—H12B	109.8	C15—N2—C14	114.5 (2)
C11—C12—H12B	109.8	C15—N3—C16	122.68 (19)
H12A—C12—H12B	108.2	C15—N3—C17	120.66 (19)
C14—C13—C16	118.3 (2)	C16—N3—C17	116.58 (19)
C14—C13—C10	113.0 (2)	C15—N4—C23	121.6 (2)
C16—C13—C10	128.7 (2)	C15—N4—H4A	119.2
N2—C14—C13	127.5 (2)	C23—N4—H4A	119.2
N2—C14—S1	120.96 (19)		
			
C6—C1—C2—C3	0.3 (5)	N3—C17—C18—C19	179.9 (2)
C1—C2—C3—C4	0.8 (4)	C17—C18—C19—C20	0.4 (4)
C2—C3—C4—C5	−1.0 (4)	C18—C19—C20—F1	179.0 (2)
C2—C3—C4—C7	179.9 (2)	C18—C19—C20—C21	−2.2 (5)
C3—C4—C5—C6	0.2 (4)	F1—C20—C21—C22	−179.0 (3)
C7—C4—C5—C6	179.3 (2)	C19—C20—C21—C22	2.2 (5)
C2—C1—C6—C5	−1.1 (4)	C20—C21—C22—C17	−0.5 (5)
C4—C5—C6—C1	0.9 (4)	C18—C17—C22—C21	−1.2 (4)
C5—C4—C7—N1	87.2 (3)	N3—C17—C22—C21	−179.9 (3)
C3—C4—C7—N1	−93.7 (3)	N4—C23—C24—C25	67.6 (5)
N1—C8—C9—C10	14.6 (4)	C9—C8—N1—C12	−49.8 (3)
N1—C8—C9—S1	−166.08 (19)	C9—C8—N1—C7	−178.5 (2)
C14—S1—C9—C10	−0.9 (2)	C11—C12—N1—C8	69.8 (3)
C14—S1—C9—C8	179.7 (2)	C11—C12—N1—C7	−162.1 (2)
C8—C9—C10—C13	−179.4 (2)	C4—C7—N1—C8	65.3 (3)
S1—C9—C10—C13	1.3 (3)	C4—C7—N1—C12	−62.4 (3)
C8—C9—C10—C11	2.0 (4)	N4—C15—N2—C14	178.2 (3)
S1—C9—C10—C11	−177.38 (19)	N3—C15—N2—C14	−1.0 (4)
C9—C10—C11—C12	15.1 (3)	C13—C14—N2—C15	1.7 (4)
C13—C10—C11—C12	−163.3 (2)	S1—C14—N2—C15	−177.2 (2)
C10—C11—C12—N1	−48.8 (3)	N2—C15—N3—C16	−0.9 (4)
C9—C10—C13—C14	−1.0 (3)	N4—C15—N3—C16	179.8 (2)
C11—C10—C13—C14	177.5 (2)	N2—C15—N3—C17	175.8 (2)
C9—C10—C13—C16	−179.1 (2)	N4—C15—N3—C17	−3.5 (4)
C11—C10—C13—C16	−0.5 (4)	O1—C16—N3—C15	−178.5 (2)
C16—C13—C14—N2	−0.4 (4)	C13—C16—N3—C15	2.2 (3)
C10—C13—C14—N2	−178.7 (2)	O1—C16—N3—C17	4.7 (3)
C16—C13—C14—S1	178.59 (18)	C13—C16—N3—C17	−174.7 (2)
C10—C13—C14—S1	0.3 (3)	C18—C17—N3—C15	101.6 (3)
C9—S1—C14—N2	179.4 (2)	C22—C17—N3—C15	−79.7 (3)
C9—S1—C14—C13	0.3 (2)	C18—C17—N3—C16	−81.5 (3)
C14—C13—C16—O1	179.2 (2)	C22—C17—N3—C16	97.2 (3)
C10—C13—C16—O1	−2.8 (4)	N2—C15—N4—C23	0.8 (5)
C14—C13—C16—N3	−1.5 (3)	N3—C15—N4—C23	−180.0 (3)
C10—C13—C16—N3	176.5 (2)	C24—C23—N4—C15	−165.6 (3)
C22—C17—C18—C19	1.2 (4)		

### Hydrogen-bond geometry (Å, º)

**Table:**

*D*—H···*A*	*D*—H	H···*A*	*D*···*A*	*D*—H···*A*
C24—H24*B*···F1^i^	0.97	2.66	3.258 (5)	121
C25—H25*A*···F1^i^	0.96	2.56	3.096 (5)	116
N4—H4*A*···F1^i^	0.86	2.65	3.423 (3)	151

Symmetry code: (i) −*x*+5/2, *y*, −*z*+1.
